# Clinical and genetic analysis of PRRT2 gene mutations in a cohort of 21 pediatric patients: a case series

**DOI:** 10.3389/fped.2026.1794255

**Published:** 2026-05-25

**Authors:** Xiaolan Zheng, Caimei Lin

**Affiliations:** Department of Neurology, Children's Hospital of Fudan University (Xiamen Branch), Xiamen Children's Hospital, Xiamen, China

**Keywords:** begign familial infantile seizures, hemiplegic migraine, mutation, paroxysmal kinesigenic dyskinesia, PRRT2 gene

## Abstract

**Background:**

The Proline-Rich Transmembrane Protein 2 (PRRT2) gene is associated with a spectrum of autosomal dominant paroxysmal disorders, including Benign Familial Infantile Epilepsy (BFIE), Paroxysmal Kinesigenic Dyskinesia (PKD), and Hemiplegic Migraine (HM). However, the correlation between specific mutation types and clinical phenotypes, particularly for rare variants, remains to be fully elucidated.

**Methods:**

We conducted a retrospective analysis of 21 pediatric patients (12 males, 9 females) with PRRT2-related disorders diagnosed at Xiamen Children's Hospital between June 2014 and December 2025. Clinical data were collected from electronic medical records. Genetic analysis was performed using family-based whole-exome sequencing. Variants were classified according to ACMG guidelines, and novel variants were assessed using bioinformatics tools (PolyPhen-2, PROVEAN, SIFT).

**Results:**

The cohort included 14 patients with BFIE, 2 with non-BFIE epilepsy, 4 with PKD, and 1 with HM. Frameshift mutations were predominant (90.5%), with c.649dupC (p.Arg217Profs*8) being the most frequent (71.4%). A novel heterozygous missense variant, c.811C > T (p.Leu271Phe), was identified in a patient with drug-resistant focal epilepsy; in silico tools predicted it to be damaging. Functional analysis revealed that the intronic variant c.880-34G > A (p.?) causes aberrant mRNA splicing, leading to a premature termination codon (p.Ser294Leufs*29). The phenotypes demonstrated a temporal succession, with BFIE manifesting in infancy (median onset 4.3 months), PKD in childhood/adolescence (mean onset 8.5 years), and HM in adolescence (onset 13 years). While most patients responded well to sodium channel blockers, one patient with a novel missense variant exhibited pharmacoresistance.

**Conclusions:**

This study expands the known mutational spectrum of PRRT2 by identifying a novel missense variant (c.811C > T) associated with refractory epilepsy and providing functional evidence for the pathogenic intronic variant c.880-34G > A. The findings highlight the time-dependent evolution of PRRT2-related phenotypes and underscore that PRRT2 mutations are not invariably associated with a benign prognosis, emphasizing the need for comprehensive genetic evaluation and personalized long-term management.

## Introduction

The Proline-Rich Transmembrane Protein 2 (PRRT2) gene, situated on chromosome 16p11.2, comprises four exons and encodes a protein consisting of 340 amino acids. This protein features an N-terminal proline-rich extracellular domain alongside two transmembrane domains located at the C-terminus. PRRT2 is broadly expressed throughout the brain, with pronounced expression observed in the cerebral cortex, cerebellar granule cells, and Purkinje cells (1, 2). Mutations in PRRT2 have been implicated in autosomal dominant disorders exhibiting a wide range of clinical phenotypes, including Benign Familial Infantile Epilepsy (BFIE), Paroxysmal Kinesigenic Dyskinesia (PKD), Infantile Convulsions with Choreoathetosis (ICCA), epilepsy, migraine, and Hemiplegic Migraine (HM) (3–5). However, the correlation between specific mutation types within PRRT2 and their associated clinical manifestations remains to be fully elucidated. In the present study, we report on a cohort of 21 patients harboring PRRT2 mutations, detailing their genotypic and phenotypic profiles. Additionally, We have identified a novel PRRT2 variant not previously reported in the literature, which may cause refractory epilepsy, thereby expanding the known mutation spectrum of this gene.

## Materials and methods

### Patients

This retrospective study analyzed the clinical and genetic characteristics of 21 patients (12 males and 9 females) diagnosed with PRRT2-related disorders at Xiamen Children's Hospital between June 2014 and December 2025. The clinical presentations within the cohort encompassed 14 cases of benign familial infantile epilepsy (BFIE), 2 cases of other epilepsy subtypes, 4 cases of paroxysmal kinesigenic dyskinesia (PKD), and 1 case of hemiplegic migraine (HM). Diagnoses were established through family-based whole-exome sequencing in conjunction with corresponding clinical features.

### Data collection

Baseline demographic and clinical information was obtained from the electronic medical record system, encompassing patient name, sex, date of birth, developmental history, and family medical history. A standardized data collection instrument was employed to document the following variables: clinical phenotype, age at onset, age at symptom remission (when applicable), seizure classification, occurrence of cluster seizures, brain MRI findings, EEG results, blood and urine tandem mass spectrometry analyses, treatment regimen, and data from the most recent follow-up, including age at follow-up, symptom status, and cognitive functioning.This article delineates three prototypical clinical syndromes, each defined by specific diagnostic criteria as detailed below.

Benign Familial Infantile Epilepsy (BFIE) is characterized by the following conditions (6): (I) a family history of seizures exhibiting a benign progression and a comparable age of onset; (II) normal developmental milestones prior to seizure onset; (III) seizure onset occurring between 3 and 10 months of age; (IV) absence of underlying disorders or neurological abnormalities; (V) seizures occurring in clusters; (VI) a benign clinical course; and (VII) normal developmental outcomes post-seizure.

Paroxysmal Kinesigenic Dyskinesia (PKD) is defined by the presence of (7): (I) identifiable kinesigenic triggers precipitating attacks; (II) brief attack duration, typically less than one minute; (III) absence of loss of consciousness or pain during episodes; (IV) exclusion of other organic pathologies and normal neurological examination findings; (V) responsiveness of attacks to phenytoin or carbamazepine treatment, if administered; and (VI) age of onset between 1 and 20 years in cases lacking a family history of PKD.

Hemiplegic Migraine (HM) diagnostic criteria include (8): (I) at least two attacks meeting criteria II and III; (II) fully reversible motor weakness and fully reversible visual, sensory, and/or speech/language symptoms; (III) the presence of at least three of the following six features: (1) gradual spread of at least one aura symptom over five minutes; (2) occurrence of two or more aura symptoms in succession; (3) duration of each individual aura symptom between 5 and 60 min; (4) unilateral manifestation of at least one aura symptom; (5) presence of at least one positive aura symptom; and (6) aura accompanied or followed within 60 min by headache; and (IV) symptoms not better explained by another diagnosis according to the International Classification of Headache Disorders, 3rd edition (ICHD-3). It is noteworthy that the term “plegic” typically denotes paralysis in most languages; however, the majority of HM attacks are characterized by motor weakness rather than complete paralysis. Additionally, motor symptoms generally resolve within 72 h, although in some patients, motor weakness may persist for several weeks.

### Genetic analysis and variant interpretation

High-throughput sequencing was conducted utilizing the Illumina NovaSeq platform. The resulting sequences were aligned to the human reference genome GRCh37/hg19, achieving a sequencing depth exceeding 20X across 96% of the targeted capture regions. Data analysis was performed using a clinical rapid diagnostic next-generation sequencing data screening system (Software Copyright Registration No.: 2022SR1373632) to identify pathogenic genes and clinically relevant genetic variants correlated with the patient's phenotype. All identified variants were classified in accordance with the Standards and Guidelines for the Interpretation of Sequence Variants established by the American College of Medical Genetics and Genomics (ACMG) (9). A standardized data collection form was employed to document details of PRRT2 variants, including cDNA alterations, amino acid substitutions, zygosity, variant classification, and inheritance patterns. Variants were cross-referenced against the ClinVar and Human Gene Mutation Database (HGMD). Novel variants, not previously reported, were subjected to further pathogenicity evaluation using bioinformatics prediction tools such as PolyPhen-2, PROVEAN, and SIFT.

### Statistics

Statistical analyses were conducted using Excel software, which facilitated the computation of medians and means. Categorical data were presented as percentages.

## Results

### Clinical characteristics

#### BFIE

The study cohort comprised fourteen individuals (patient 1–14) diagnosed with BFIE, including six males and eight females (Table 1). The onset of seizures occurred between two and seven months of age, with a median onset age of 4.3 months. Seizure remission was observed between three and twelve months, with a median age of 7.5 months. Thirteen patients experienced focal motor seizures, whereas one patient presented with generalized tonic-clonic seizures. A distinctive feature across all cases was the presence of seizure clusters. Cranial magnetic resonance imaging (MRI) revealed no significant abnormalities in any patient, and no developmental delays were detected. Screening via blood and urine tandem mass spectrometry yielded normal results for all participants. Electroencephalographic (EEG) evaluations indicated normal patterns in ten patients, however, four exhibited interictal spike-and-slow wave complexes. Specifically, patient 1 showed sporadic spike-and-slow wave discharges localized to the bilateral anterior frontal regions; patient 4 demonstrated a limited number of such discharges in the left middle and posterior temporal regions; and patient 5 and patient 7 presented with a high frequency of spike-and-slow wave complexes in the bilateral parietal and occipital regions. Regarding antiepileptic drug (AED) management, five patients experienced infrequent seizures and thus did not receive pharmacological treatment. Eight patients were treated with monotherapy: five with oxcarbazepine, two with valproate, and one with levetiracetam. Additionally, one patient required combination therapy with levetiracetam and lacosamide to achieve satisfactory seizure control. At the most recent follow-up, conducted when patients were between two and eight years of age, all remained seizure-free and demonstrated cognitive performance within normative limits on standardized intelligence assessments.

**Table 1:** Clinical characteristics of 21 patients with PRRT2 gene mutation.

										Status at last follow-up			
Patient,Gender	Phenotype	Age of onset	Age of offset	Seizure Type	Cluster Seizures	Cranial MRI	EEG	MS/MS	Medication			Intellectual Level	
										Age	Symptom		
												Assessment tool	IQ/DQ
P1,F	BFIE	3m	3m	Focal Motor	YES	Normal	Frontal	Normal	VPA	5y	Asymptomatic	WISC	90
P2, M	BFIE	2m	3m	GTCS	YES	Normal	Normal	Normal	VPA	5y	Asymptomatic	WISC	105
P3, F	BFIE	6m	12m	Focal Motor	YES	Normal	Normal	Normal	None	7y	Asymptomatic	WISC	100
P4, F	BFIE	6m	12m	Focal Motor	YES	Normal	Temporal	Normal	LEV,LCM	3y	Asymptomatic	GDS	99
P5, M	BFIE	4m	5m	Focal Motor	YES	Normal	Parietal, Occipital	Normal	OXC	2y	Asymptomatic	GDS	95
P6, F	BFIE	6m	11m	Focal Motor	YES	Normal	Normal	Normal	None	6y	Asymptomatic	WISC	105
P7, M	BFIE	3m	3m	Focal Motor	YES	Normal	Parietal,Occipital	Normal	OXC	2y	Asymptomatic	GDS	99
P8, F	BFIE	7m	8m	Focal Motor	YES	Normal	Normal	Normal	None	2y	Asymptomatic	GDS	88
P9, M	BFIE	3m	12m	Focal Motor	YES	Normal	Normal	Normal	OXC	7y	Asymptomatic	WISC	104
P10, F	BFIE	4m	6m	Focal Motor	YES	Normal	Normal	Normal	OXC	8y	Asymptomatic	WISC	110
P11, F	BFIE	3m	7m	Focal Motor	YES	Normal	Normal	Normal	OXC	6y	Asymptomatic	WISC	110
P12, M	BFIE	5m	8m	Focal Motor	YES	Normal	Normal	Normal	None	4y	Asymptomatic	WISC	100
P13, F	BFIE	3m	9m	Focal Motor	YES	Normal	Normal	Normal	None	4y	Asymptomatic	WISC	108
P14, M	BFIE	6m	6m	Focal Motor	YES	Normal	Normal	Normal	LEV	2y	Asymptomatic	GDS	90
						Normal		Normal			Asymptomatic		
P15, M	epilepsy	4m	13y	Focal Motor	YES	Normal	Normal	Normal	LEV,OXC	14y	Asymptomatic	WISC	105
P16, F	epilepsy	6y	On going	Focal Motor	YES	Normal	Central, Parietal, Temporal	Normal	LEV,VPA,OXC,LCM,PER	8y	1-2/month	WISC	8y
P17, M	PKD	8y	11y	/	/	Normal	Normal	Normal	OXC	13y	Asymptomatic	WISC	100
						Normal		Normal			Asymptomatic		
P18, M	PKD	8y	9y	/	/	Normal	Normal	Normal	OXC	14y	Asymptomatic	WISC	102
P19, M	PKD	9y	11y	/	/	Normal	Normal	Normal	OXC	14y	Asymptomatic	WISC	89
P20, M	PKD	9y	12y	/	/	Normal	Normal	Normal	OXC	15y	Asymptomatic	WISC	96
P21, M	HM	13y	On going	/	/	Normal	Normal	Normal	None	14y	1/3–4 m	WISC	102

F, female; M, male; m, month; y, year, BFIE, benign familial infantile epilepsy; PKD, paroxysmal kinesigenic dyskinesia; HM, hemiplegic migraine; MS/MS, Tandem Mass Spectrometry; WISC, Wechsler Intelligence Scale for Children; GDS, Gesell Developmental Schedules; VPA, valproate; LEV, levetiracetam; LCM, lacosamide; OXC, oxcarbazepine; PER, perampanel.

#### Epilepsy

Patient 15 and patient 16 diagnosed with epilepsy, excluding those with benign familial infantile epilepsy (non-BFIE) as outlined in Table 1, both presented with focal motor seizures. The patient 15 experienced seizure onset at four months of age, with normal findings on cranial MRI and EEG. This patient exhibited typical cognitive development and achieved seizure remission by the age of 13 after receiving combination therapy with levetiracetam and oxcarbazepine. The patient 16 developed drug-resistant epilepsy, with seizures beginning at six years of age. Clinically, this individual exhibited focal seizures accompanied by mild developmental delay. Cranial MRI results were unremarkable, whereas EEG demonstrated background slowing characterized by a 5–7 Hz theta rhythm during wakefulness. Sleep EEG recordings revealed frequent spike-and-slow wave discharges localized to the left central, parietal, middle temporal, and posterior temporal regions. Despite sequential treatment with five antiseizure medications-levetiracetam, valproate, oxcarbazepine, lacosamide, and perampanel-administered according to established protocols, this patient continued to experience sporadic focal motor seizures.

#### PKD

Four individuals (patient 17–20) diagnosed with PKD, as detailed in Table 1, exhibited a mean age of symptom onset at 8.5 years. Their clinical presentations involved abrupt episodes of abnormal posturing and involuntary limb twisting movements, precipitated by voluntary motor activity. Each episode persisted for approximately one minute and resolved spontaneously without any loss of consciousness. Neuroimaging evaluations, includingMRI and EEG, yielded normal results. Furthermore, all patients demonstrated cognitive development consistent with their chronological age. Symptom management was effectively achieved with low-dose oxcarbazepine, resulting in rapid clinical improvement.

#### HM

Patient 21 diagnosed with HM (Table 1) exhibited symptom onset at 13 years of age. The clinical presentation comprised a left frontal headache accompanied by numbness localized to the right side of the tongue and the distal segment of the right limb, micropsia, and 5 to 6 episodes of emesis containing gastric contents. Each episode persisted for 3 to 4 h and resolved spontaneously following a period of rest. The patient's monozygotic twin brother reported a similar clinical course, with symptom onset at 11 years of age, characterized by sudden unilateral headache, contralateral limb numbness, and blurred vision. These episodes lasted several hours and recurred approximately three times per year. Both twins exhibited normal results on cranial MRI, EEG, and developmental assessments. Neither individual was undergoing pharmacological treatment, and both continued to experience intermittent attacks.

#### PRRT2 gene mutations

**Figure 1:**
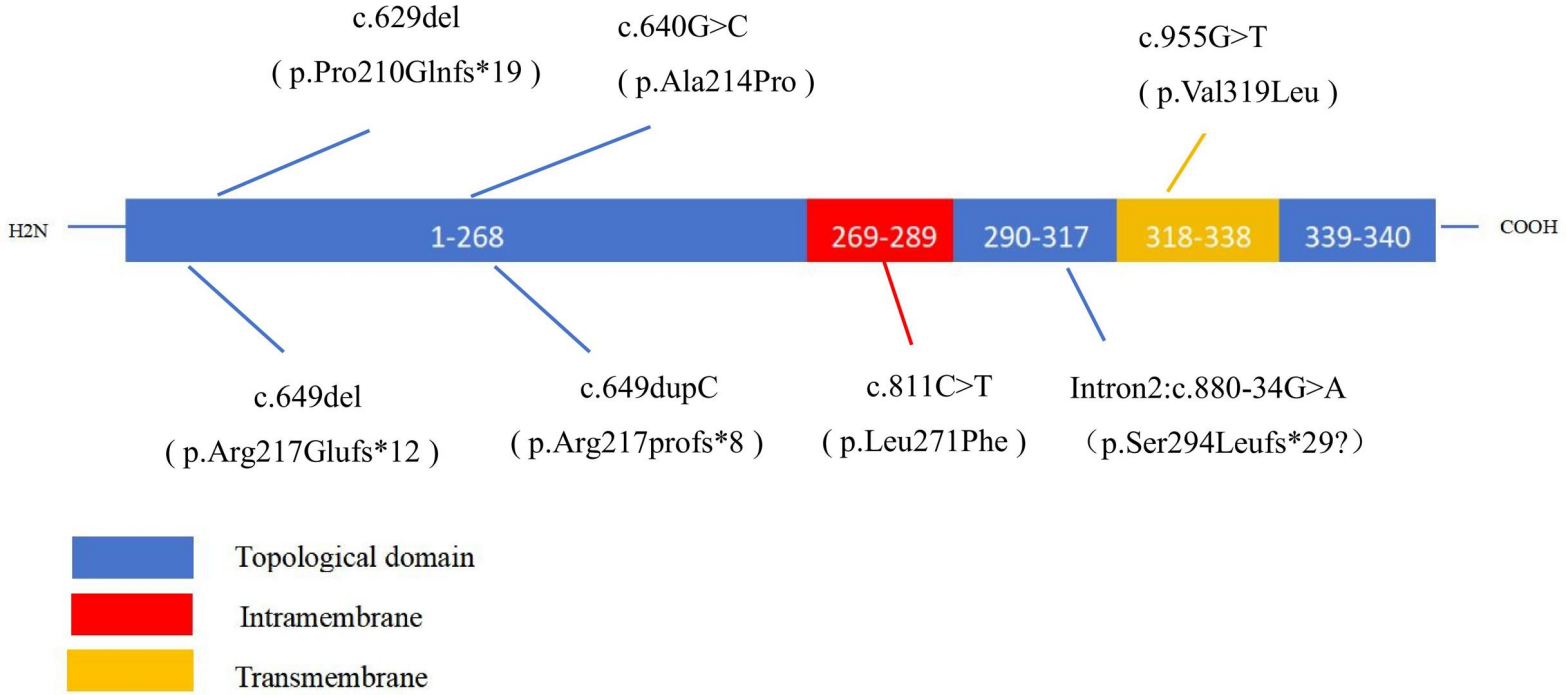
Positions of amino acid alterations caused by PRRT2 variants in this study.

Among the cohort of 21 patients, nine distinct genetic variants were identified (Figure 1; Table 2). Frameshift mutations predominated, being present in 19 patients (90.5%). Specifically, the c.649dupC (p.Arg217Profs*8) variant was observed in 15 patients (71.4%), while c.629delC (p.Pro210Glnfs*19) and c.649delC (p.Arg217Glufs*12) were each detected in one patient (4.8%). Intronic variants were detected in two patients (9.5%). One such variant, Intron3: c.1013-29del, is documented as pathogenic in the Human Gene Mutation Database (HGMD). This variant, situated within the intron adjacent to exon 3, is predicted to induce out-of-frame transcription and a delayed stop codon, thereby producing an elongated transcript (10). Notably, this patient also exhibited a deletion mutation of the PRRT2 gene on the contralateral chromosome. The second intronic variant, Intron2: c.880-34G > A (p.?), also classified as pathogenic in HGMD, was investigated via RT-PCR analysis of RNA extracted from lymphocytes of the patient and his affected mother. This analysis revealed that the mutant mRNA was extended by 36 nucleotides relative to the wild-type transcript, suggesting that this variant may result in out-of-frame transcription and a premature termination codon (p.Ser294Leufs*29). Missense mutations were identified in two patients (9.5%). One patient exhibited compound heterozygous variants: c.955G > T (p.Val319Leu) and c.640G > C (p.Ala214Pro). The other patient carried a heterozygous c.811C > T (p.Leu271Phe) mutation, inherited from an asymptomatic mother. This variant has not been previously reported in the ClinVar or HGMD databases. In silico analyses predicted this mutation to be “probably damaging” according to PolyPhen-2 (score 0.997), “deleterious” by PROVEAN (score −3.33), and “damaging” as per SIFT (score 0.004). Clinically, this patient presented with refractory focal epilepsy.

**Table 2:** Mutational characteristics of 21 patients with PRRT2 gene mutation.

Patient, Gender	Phenotype	Varient	Protein	Parental derivation/Family members with the variant	gnomAD	ACMG Classication		FH+	
							Relationship		Phenotype
P1,F	BFIE	c.629del	p.Pro210Glnfs*19	Mother/Sister（Het）	NA	P	Mother		BFIE
P2, M	BFIE	c.649del	p.Arg217Glufs*12	De novo	0.0004	P		None	
P3, F	BFIE	c.649dupC	p.Arg217profs*8	De novo	0.0003	P		None	
P4, F	BFIE	c.649dupC	p.Arg217profs*8	Mother	0.0003	P		None	
P5, M	BFIE	c.649dupC	p.Arg217profs*8	De novo	0.0003	P		None	
P6, F	BFIE	c.649dupC	p.Arg217profs*8	De novo	0.0003	P		None	
P7, M	BFIE	c.649dupC	p.Arg217profs*8	Mother	0.0003	P		None	
P8, F	BFIE	c.649dupC	p.Arg217profs*8	De novo	0.0003	P		None	
P9, M	BFIE	c.649dupC	p.Arg217profs*8	De novo	0.0003	P		None	
P10, F	BFIE	c.649dupC	p.Arg217profs*8	De novo	0.0003	P		None	
P11, F	BFIE	c.649dupC	p.Arg217profs*8	De novo	0.0003	P		None	
P12, M	BFIE	c.649dupC	p.Arg217profs*8	De novo	0.0003	P		None	
P13, F	BFIE	c.649dupC	p.Arg217profs*8	De novo	0.0003	P		None	
P14, M	BFIE	Intron3:c.1013-29del	p.?	Mother/Brother（Het）	<0.0001	LP	Mother		BFIE
		16p11.2 del	524.96kbp	Father	/	P			
P15, M	epilepsy	c.649dupC	p.Arg217profs*8	De novo	0.0003	P		None	
P16, F	epilepsy	c.811C > T	p.Leu271Phe	Mother	NA	VUS		None	
P17, M	PKD	c.955G > T	p.Val319Leu	Father	0.0052	VUS	Father		PKD
		c.640G > C	p.Ala214Pro	Father	0.0001	VUS			
P18, M	PKD	c.649dupC	p.Arg217profs*8	Mother	0.0003	P	Mother		PKD
P19, M	PKD	c.649dupC	p.Arg217profs*8	De novo	0.0003	P		None	
P20, M	PKD	c.649dupC	p.Arg217profs*8	De novo	0.0003	P		None	
P21, M	HM	Intron2:c.880-34G > A	p.Ser294Leufs*29?	De novo/Brother(Het)	NA	P	Brother		HM

F, female; M, male; NA, not available; P, pathogenic; LP, likely pathogenic; VUS, variant of uncertain significance; BFIE, benign familial infantile epilepsy; PKD, paroxysmal kinesigenic dyskinesia; HM, hemiplegic migraine; FH+, positive family history.

*A stop codon (translation termination).

## Discussion

PRRT2 (proline-rich transmembrane protein 2) has been firmly established as a causative gene for a spectrum of paroxysmal neurological disorders, primarily benign familial infantile epilepsy (BFIE), infantile convulsion and choreoathetosis (ICCA),paroxysmal kinesigenic dyskinesia (PKD), and hemiplegic migraine (HM) (11–14). Since its initial identification in 2011 (1), an expanding body of evidence has demonstrated that PRRT2 mutations account for a significant proportion of families with these autosomal dominant conditions, with frameshift mutations—particularly the recurrent c.649dupC (p.Arg217Profs*8)—representing the predominant mutation type (11, 15). The encoded protein plays a critical role in regulating synaptic exocytosis through interaction with SNAP25, and loss-of-function mutations are believed to impair neuronal transmitter release, thereby increasing neuronal excitability and predisposing individuals to paroxysmal events (16). PRRT2 also engages with other synaptic proteins, such as vesicle-associated membrane protein 2 (VAMP2) and synaptotagmins 1 and 2. Through these interactions, it modulates voltage-gated ion channels and participates in Ca^2^⁺-regulated neurotransmitter release (17, 18). Furthermore, PRRT2 has been shown to negatively regulate voltage-gated Nav1.2 and Nav1.6 channels by modulating their voltage-dependent inactivation and recovery from inactivation (19).Despite the phenotypic spectrum continues to expand, the relationship between specific mutation types—particularly rare variants and non-coding alterations—and clinical heterogeneity remains incompletely understood, and functional evidence supporting pathogenicity for many identified variants is still lacking (11).

In the present study, we performed comprehensive clinical and genetic analyses on a cohort of 21 Chinese pediatric patients harboring PRRT2 gene mutations, Our cohort encompassed four phenotypic spectrum of PRRT2-related disorders, including BFIE (*n* = 14), non-BFIE epilepsy (*n* = 2), PKD (*n* = 4), and HM (*n* = 1), thereby recapitulating the well-established clinical heterogeneity associated with this gene. Beyond confirming the prevalence of common frameshift mutations (c.649dupC accounting for 71.4% of our cases), we identified several noteworthy genetic findings that extend the current understanding of PRRT2-related pathogenesis. First, we characterized a novel missense variant, c.811C > T (p.Leu271Phe), in a patient with drug-resistant focal epilepsy, which has not been previously documented in public databases. This variant is located in a highly conserved region of the PRRT2 protein and is completely absent from the gnomAD database, indicating its extreme rarity in the general population. Multiple in silico tools consistently predicted its deleterious nature: PolyPhen-2 scored 0.997 (“probably damaging”), PROVEAN scored −3.33 (“deleterious”), and SIFT scored 0.004 (“damaging”). Critically, the proband carrying this variant presented with drug-resistant focal epilepsy, exhibiting a poor response to combination therapy with five antiseizure medications (levetiracetam, valproate, oxcarbazepine, lacosamide, and perampanel). This phenotype markedly diverges from the typically benign, self-limiting course associated with PRRT2 mutations (20, 21). Although the variant was inherited from an asymptomatic carrier mother—illustrating the phenomenon of incomplete penetrance in PRRT2-related disorders (22), the severe clinical manifestation in the proband strongly suggests that this missense alteration may lead to more pronounced neurological deficits through a pathogenic mechanism distinct from classical loss-of-function frameshift mutations (3). We hypothesize that potential mechanisms include genetic modifiers (such as co-segregating variants in other ion channel or synaptic genes), environmental factors (e.g., triggering events like infections or stress), as well as somatic mosaicism or differences in epigenetic regulation. Furthermore, different mutation types may exert their pathogenic effects through distinct molecular mechanisms: classical frameshift mutations typically result in haploinsufficiency, whereas missense mutations may interfere with wild-type PRRT2 protein function through dominant-negative effects or acquire novel toxic functions, thereby leading to more severe neurological deficits (23). Second, we provided critical functional evidence for the intronic variant c.880-34G > A. Although this variant is documented as pathogenic in the Human Gene Mutation Database (HGMD) (24), direct functional evidence supporting its pathogenicity has been lacking. Through RT-PCR analysis of RNA extracted from peripheral blood lymphocytes of the patient and his affected mother, we demonstrated that the mutant mRNA was extended by 36 nucleotides compared to the wild-type transcript. Subsequent sequencing confirmed that this extension resulted from the aberrant retention of intronic sequences, leading to an out-of-frame open reading frame and the generation of a premature termination codon (p.Ser294Leufs*29). This finding definitively establishes that this intronic variant exerts its pathogenic effect by disrupting normal mRNA splicing, rather than representing a benign polymorphism. Notably, this patient also carried a deletion mutation in the PRRT2 gene on the contralateral allele, constituting a compound heterozygous state. This finding contrasts with previous studies, which have suggested that biallelic mutations are more prone to lead to phenotypic worsening (25). However, this patient presented with only a BFIE phenotype, further highlighting the heterogeneity of PRRT2 gene mutations.

This cohort encompassed the phenotypic spectrum of PRRT2-related disorders—BFIE (*n* = 14), PKD (*n* = 4), and HM (*n* = 1)—providing a unique perspective for understanding the time-dependent evolutionary patterns of diseases associated with this gene (3). Notably, these three conditions exhibited a clear temporal succession in terms of age at onset: BFIE typically manifested during infancy (median onset age 4.3 months in this cohort), PKD predominantly emerged from childhood to adolescence (mean onset age 8.5 years in this cohort), while HM tended to present during adolescence to early adulthood (13 years in this cohort). This temporal succession pattern suggests that PRRT2 mutation-related paroxysmal phenotypes may not represent isolated events but rather the dynamic expression of the same molecular defect at different developmental stages (26).The potential mechanism underlying this phenomenon may be related to the functional development-dependent characteristics of the PRRT2 protein (1). PRRT2 expression in the nervous system exhibits developmental regulation, with high expression in the cortex and hippocampus during early developmental stages, participating in synaptic formation and network construction of immature neurons (27). As the central nervous system matures, its expression gradually becomes concentrated in motor regulatory regions such as the basal ganglia and cerebellum (28). Consequently, cortical hyperexcitability during infancy predisposes individuals to epileptic seizures, whereas following functional maturation of the basal ganglia during childhood, paroxysmal kinesigenic dyskinesia emerges as the predominant phenotype (29). The occurrence of migraine may reflect increased susceptibility of the trigeminovascular system to PRRT2 functional defects after adolescence (30). The pair of monozygotic twins in our cohort, both presenting with HM with closely aligned ages of onset (11 and 13 years), further supports the potent regulatory role of genetic background in phenotypic expression. From a clinical practice perspective, this time-dependent evolutionary pattern carries important implications, Integrating BFIE, PKD, and HM into a conceptual framework of “PRRT2-related paroxysmal disorder spectrum” rather than viewing them as isolated disease entities will facilitate comprehensive life-cycle management for these patients.

PRRT2-related disorders generally carry a favorable prognosis (26, 31, 32), however, the differential treatment responses observed in this cohort provide important insights for clinical management. Tailored treatment strategies should be developed based on specific phenotypes. For patients with BFIE, all eight individuals in this cohort who received monotherapy (oxcarbazepine in five, valproate in two, and levetiracetam in one) achieved seizure freedom, confirming the good responsiveness of PRRT2-related epilepsy to sodium channel blockers (33). Notably, five BFIE patients with sparse seizures did not receive any antiseizure medication and still achieved spontaneous remission, suggesting that for BFIE patients with low seizure frequency and absence of status epilepticus, clinical observation rather than immediate pharmacological intervention may be a reasonable option—a strategy that avoids unnecessary drug exposure. For PKD patients, all four cases in this cohort exhibited prompt response to low-dose oxcarbazepine with complete resolution of symptoms, further establishing sodium channel blockers as first-line therapy for PKD, with effective seizure control achievable at low doses (34, 35). However, the case of drug-resistant epilepsy in this cohort serves as a critical cautionary note. Despite sequential treatment with five antiseizure medications (levetiracetam, valproate, oxcarbazepine, lacosamide, and perampanel) according to established protocols, this patient continued to experience sporadic focal motor seizures. This phenomenon indicates that PRRT2 mutations cannot be simplistically equated with a “benign prognosis,” and a minority of patients may develop drug-resistant epilepsy. It is noteworthy that this patient showed suboptimal response to sodium channel blockers (oxcarbazepine and lacosamide), contradicting the classical therapeutic expectations for PRRT2-related disorders. Potential explanations include: this missense mutation may cause disease through mechanisms distinct from frameshift mutations; there may be other genetic modifiers affecting drug target sensitivity; or following disease progression to a certain stage, neural network remodeling may lead to decreased responsiveness to sodium channel blockers.

Several limitations of this study should be considered when interpreting the results. First, as a single-center case series, the sample size is relatively limited (21 patients). Although this sample size is acceptable given the rarity of PRRT2-related disorders, it may affect the comprehensive capture of rare genotype-phenotype associations and precludes statistically meaningful subgroup analyses. Second, genetic analysis primarily targeted the coding regions and flanking intronic sequences of the PRRT2 gene, without performing whole-exome or whole-genome sequencing. Consequently, we cannot exclude potential contributions from other modifier genes or digenic inheritance to the phenotypic heterogeneity observed in our cohort. Third, regarding functional validation, although we provided crucial mRNA-level evidence for the intronic variant c.880-34G > A through RT-PCR, we were unable to conduct further studies on protein expression quantification, subcellular localization, or electrophysiological function. Therefore, direct evidence for the precise molecular pathogenic mechanisms of this variant and the novel missense mutation c.811C > T remains lacking.

## Conclusion

Through comprehensive clinical and genetic analysis of 21 Chinese pediatric patients, this study expands the mutational spectrum of the PRRT2 gene, reports for the first time the novel missense mutation c.811C > T, and provides RNA-level functional evidence for the intronic variant c.880-34G > A. It reveals the complete phenotypic spectrum of PRRT2-related disorders—from BFIE and PKD to HM—and their time-dependent evolutionary patterns. Of particular importance, we identified the non-classical phenotype of drug-resistant epilepsy and observed incomplete penetrance, indicating that PRRT2 mutations cannot be simplistically equated with a benign prognosis. These findings underscore the necessity of long-term follow-up, comprehensive genetic evaluation, and personalized treatment for PRRT2-related disorders, and lay the foundation for future investigations into the molecular mechanisms underlying phenotypic heterogeneity.

## Data Availability

The original contributions presented in the study are publicly available. This data can be found here: https://www.ncbi.nlm.nih.gov/bioproject/PRJNA1467135/ Accession: PRJNA1467135.
